# Supplementary material for: Repeated Disuse Atrophy Imprints a Molecular Memory in Skeletal Muscle: Transcriptional Resilience in Young Adults and Susceptibility in Aged Muscle
Source: Adv Sci (Weinh). 2026 Feb 25;13(23):e22726. doi: 10.1002/advs.202522726 (PMC13104094; doi:10.1002/advs.202522726)
Supplement: Supplementary file 1 — Supporting File 1: advs74388‐sup‐0001‐Figure S1.pdf. [file ADVS-13-e22726-s004.pdf]

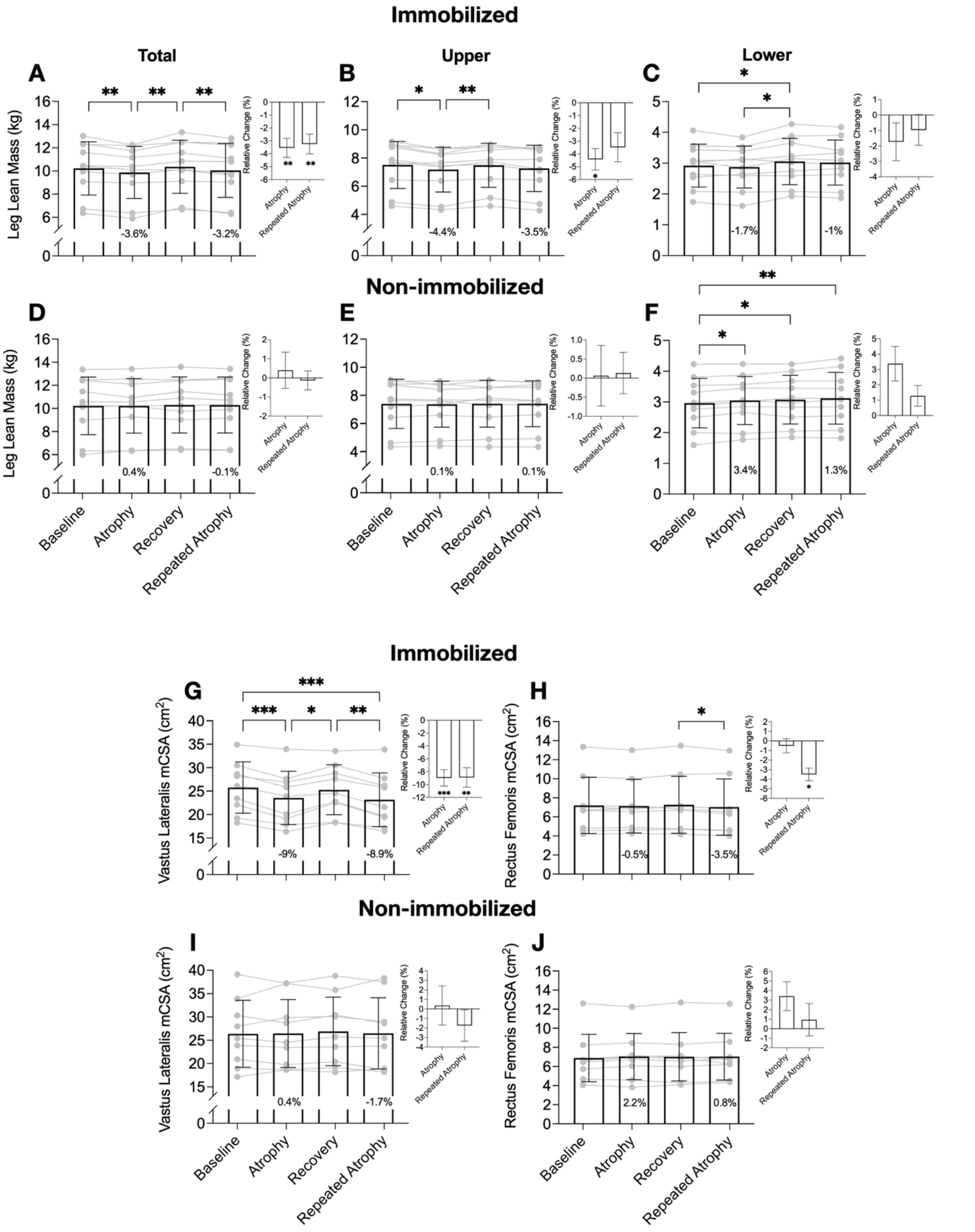

Figure S1. Human leg lean mass of the immobilised (**A-C**) and non-immobilised (**D-F**) limbs, including measurements of total (**A & D**), upper (**B & E**) and lower (**C & F**) leg lean mass. Bar graphs depict relative change (%) from the previous timepoint (i.e., atrophy versus baseline, repeated atrophy versus recovery). N = 10. Human mCSA of the immobilised (**G-H**) and non-immobilised (**I-J**) limbs, for *VL* (**G & I**) and *RF* (**H & J**) muscles. Bar graphs depict relative change (%) from the previous time point (i.e., atrophy versus baseline, repeated atrophy versus recovery). N = 9. *$p \leq 0.05$, **$p \leq 0.01$, ***$p \leq 0.001$.
