# Supplementary material for: Repeated Disuse Atrophy Imprints a Molecular Memory in Skeletal Muscle: Transcriptional Resilience in Young Adults and Susceptibility in Aged Muscle
Source: Adv Sci (Weinh). 2026 Feb 25;13(23):e22726. doi: 10.1002/advs.202522726 (PMC13104094; doi:10.1002/advs.202522726)
Supplement: Supplementary file 2 — Supporting File 2: advs74388‐sup‐0002‐Figure S2.pdf. [file ADVS-13-e22726-s006.pdf]

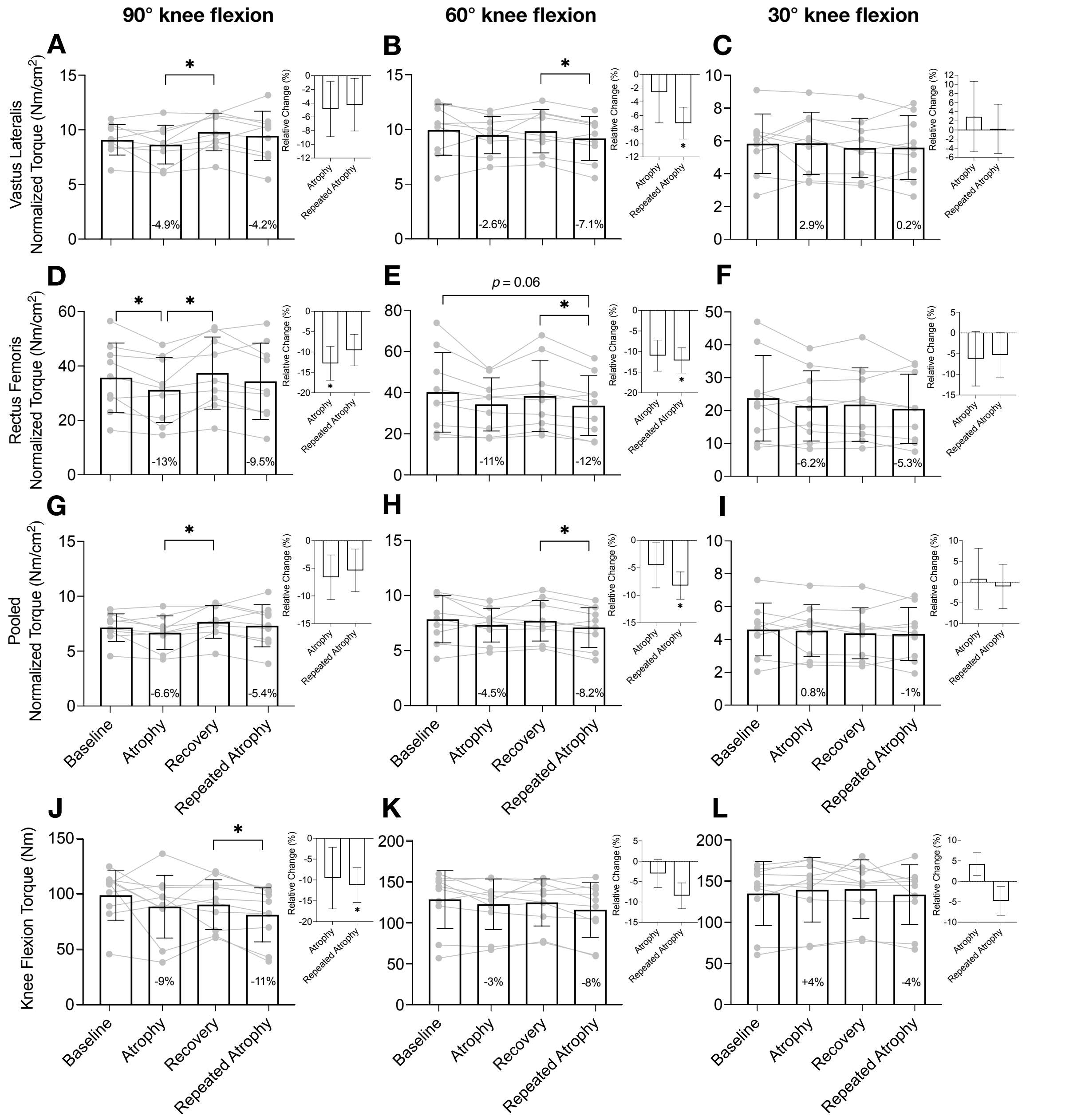

Figure S2. Human specific knee extension strength (**A-I**). Knee extension torque normalised to mCSA of the *VL* (**A-C**), *RF* (**D-F**) and sum of *VL* + *RF* (**G-I**) at 90° (**A/D/G**), 60° (**B/E/H**) and 30° (**C/F/I**) knee flexion. Knee flexion torque at 90° (**J**), 60° (**K**) and 30° (**L**) knee flexion. Bar graphs depict relative change (%) from the previous time point (i.e., atrophy versus baseline, repeated atrophy versus recovery). $N = 10$. $*p \leq 0.05$.
