# Supplementary material for: Repeated Disuse Atrophy Imprints a Molecular Memory in Skeletal Muscle: Transcriptional Resilience in Young Adults and Susceptibility in Aged Muscle
Source: Adv Sci (Weinh). 2026 Feb 25;13(23):e22726. doi: 10.1002/advs.202522726 (PMC13104094; doi:10.1002/advs.202522726)
Supplement: Supplementary file 3 — Supporting File 3: advs74388‐sup‐0003‐Figure S3.pdf. [file ADVS-13-e22726-s005.pdf]

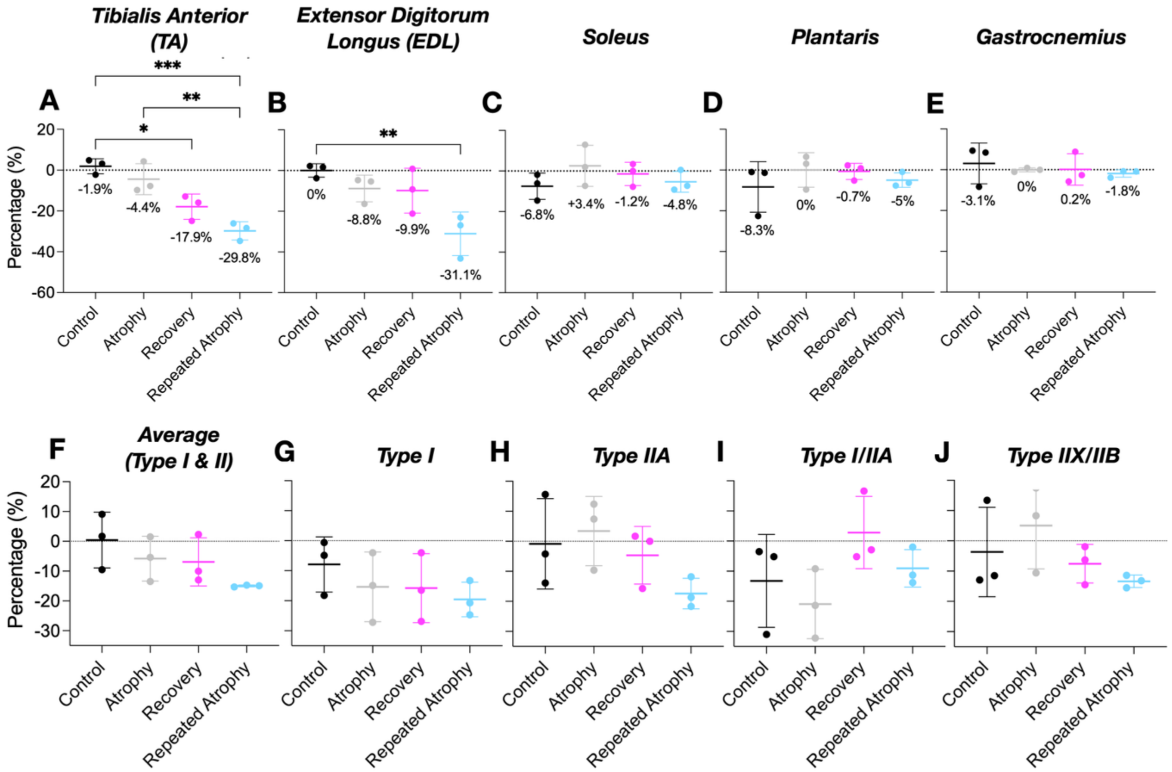

Figure S3. Muscle weights of *TA* (**A**), *EDL* (**B**), *soleus* (**C**), *plantaris* (**D**) and *gastrocnemius* (**E**) muscles. Muscle weights presented as % change of the left surgical versus right non-surgical control limb. Relative fCSA (left/surgical % of right/non-surgical limb normalized to body weight) for each fiber type (**F**) - average type I & II, (**G**) - type I, (**H**) - type IIA, (**I**) - type I/IIA and (**J**) - type IIX/IIB. Control consists of pooled right contralateral control limbs across all conditions (n = 12). $*p \le 0.05$, $**p \le 0.01$, $***p \le 0.001$.
