# Supplementary material for: Repeated Disuse Atrophy Imprints a Molecular Memory in Skeletal Muscle: Transcriptional Resilience in Young Adults and Susceptibility in Aged Muscle
Source: Adv Sci (Weinh). 2026 Feb 25;13(23):e22726. doi: 10.1002/advs.202522726 (PMC13104094; doi:10.1002/advs.202522726)
Supplement: Supplementary file 4 — Supporting File 4: advs74388‐sup‐0004‐Figure S4.pdf. [file ADVS-13-e22726-s008.pdf]

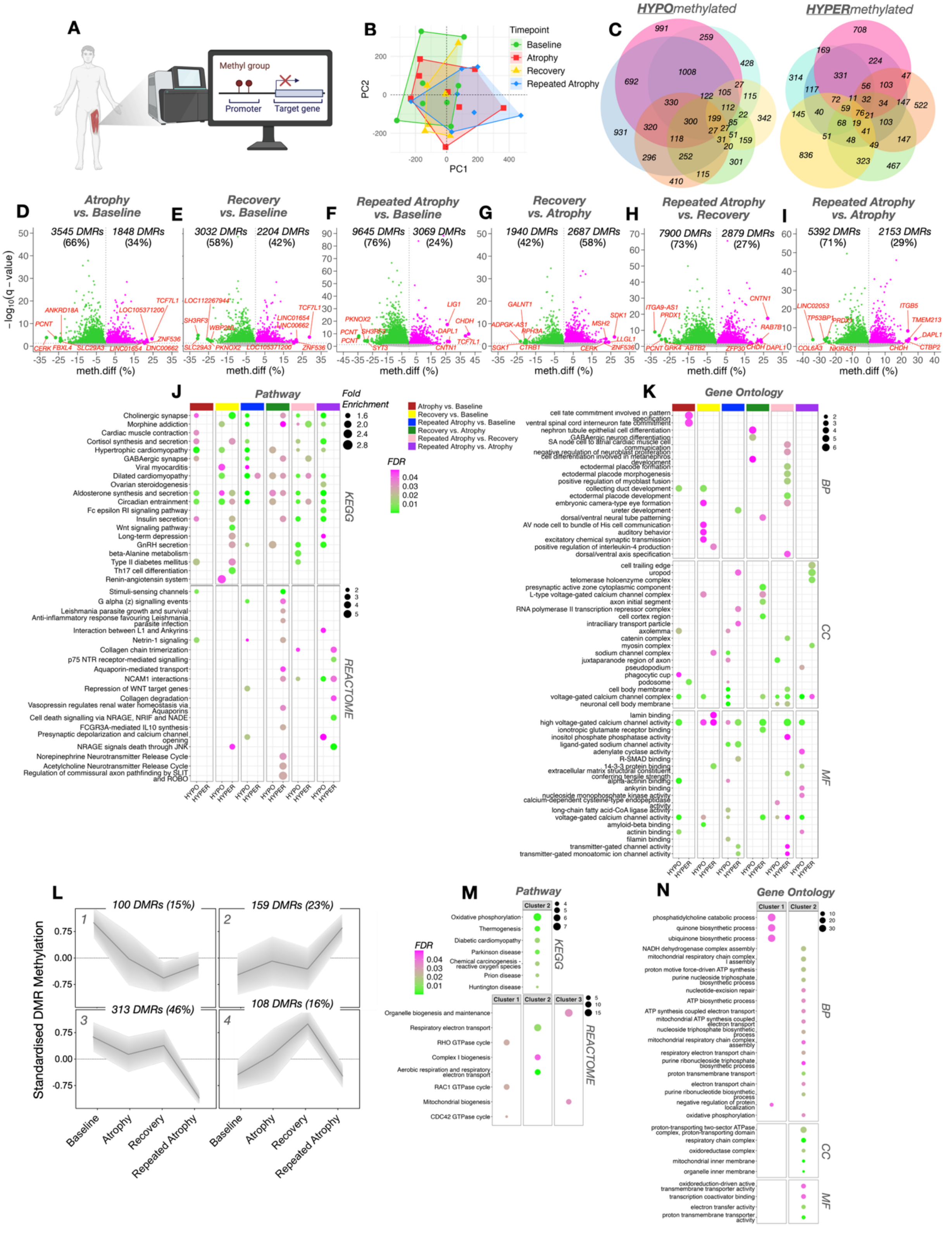

Figure S4. (**A**) Schematic depicting the genome-wide DNA methylation (via RRBS) response to repeated disuse in adult human skeletal muscle (created with BioRender.com). (**B**) Principal component analysis (PCA) including clustering by time point. (**C**) Venn diagrams depicting the number of unique and

common genes with associated HYPO and HYPER differential methylated regions (DMRs). (**D-I**) Volcano plots depicting the number of DMRs that were either HYPO- (green) or HYPER-methylated (magenta). Gene symbols within volcano plots represent the top 5 DMRs according to the greatest methylation difference (meth.diff %) with q-value (FDR) ≤ 0.05. Over-representation analysis (ORA) of the top 20 enriched (**J**) pathway (KEGG, top 4 panels; REACTOME, bottom 4 panels) and (**K**) gene ontology (GO) terms for both HYPO- and HYPER-methylated DMRs across all comparisons ("Atrophy vs. Control", "Recovery vs. Control", "Repeated Atrophy vs. Control", "Recovery vs. Atrophy", "Repeated Atrophy vs. Recovery"). (**L**) Self-Organizing Maps (SOM) gene clustering analysis of 608 DMRs by gene symbol (that were significantly differentially methylated in at least one comparison) revealed most DMRs were either HYPER- (Cluster 2) or HYPO-methylated (Cluster 3) after atrophy, return to baseline levels following recovery with larger changes (in the same direction) after repeated atrophy. Pathway (**M**) and (**N**) gene ontology (GO) enrichment analysis of these SOM cluster profiles revealed most enriched terms were related to oxidative and energy metabolism and mitochondrial biogenesis / function. BP = biological process, CC = cellular component, MF = molecular function.
