# Supplementary material for: Repeated Disuse Atrophy Imprints a Molecular Memory in Skeletal Muscle: Transcriptional Resilience in Young Adults and Susceptibility in Aged Muscle
Source: Adv Sci (Weinh). 2026 Feb 25;13(23):e22726. doi: 10.1002/advs.202522726 (PMC13104094; doi:10.1002/advs.202522726)
Supplement: Supplementary file 5 — Supporting File 5: advs74388‐sup‐0005‐Figure S5.pdf. [file ADVS-13-e22726-s003.pdf]

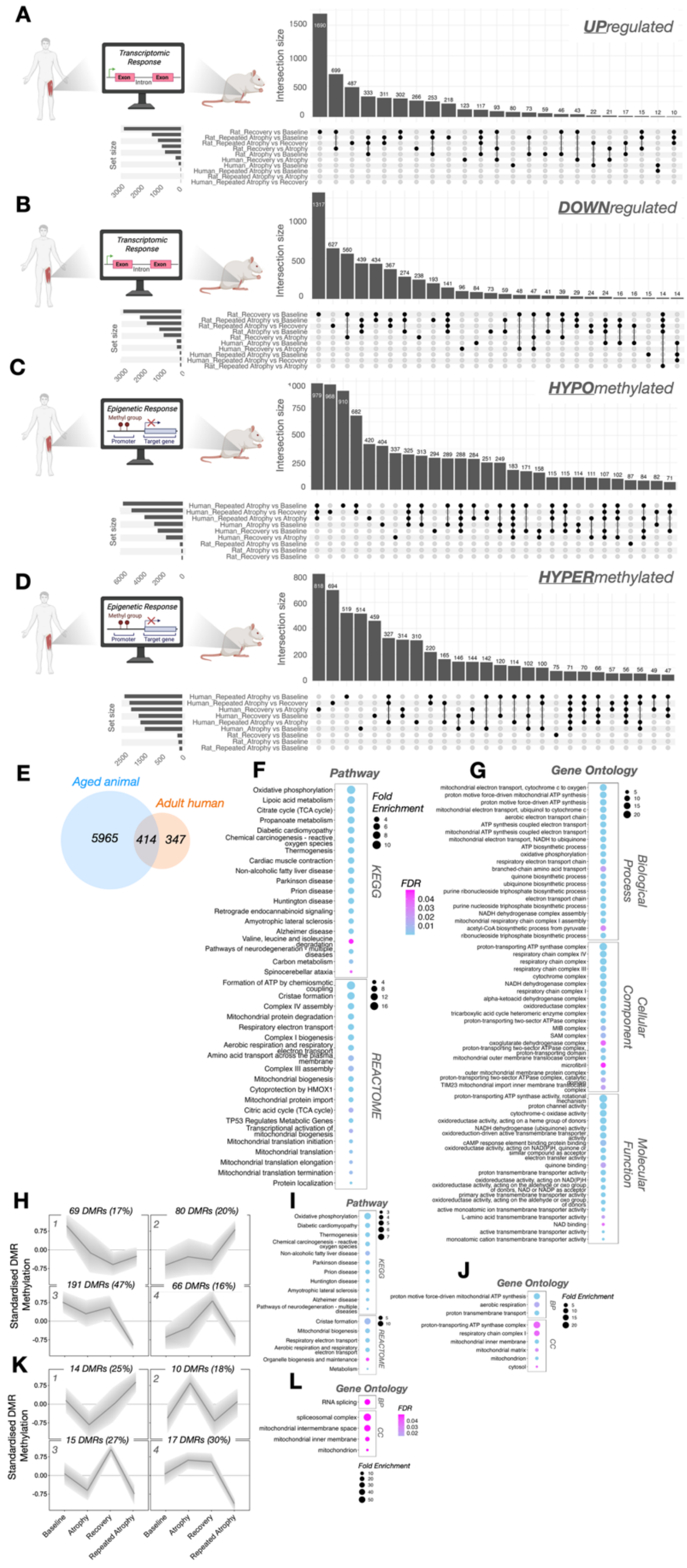

Figure S5. Comparison of the transcriptomic (**A & B**) and epigenetic (**C & D**) responses to repeated disuse atrophy in young adult human and age rat skeletal muscle across all 6 pairwise comparisons. Identifying several common and unique UP-regulated (**A**) and DOWN-regulated (**B**) DEGs as well as HYPO-methylated (**C**) and HYPER-methylated (**D**) DMRs within each pairwise comparison. (**E**) Venn diagram showing all genes significantly differentially

expressed in at least one pairwise comparison in young adult humans (761 DEGs) and aged rats (6379 DEGs). A total of 414 DEGs were shared between models, indicating that more than half of all human skeletal muscle DEGs (54%) were also differentially expressed in aged rat skeletal muscle after atrophy, recovery and repeated atrophy. Pathway (**F**) and GO (**G**) analyses demonstrated these genes were related to oxidative metabolism, energy metabolism and mitochondrial function. (**H**) SOM temporal clustering profiling analysis identified 406 DMRs on 189 genes (out of 414 DEGs) in adult human muscle, with genes related to oxidative metabolism and mitochondrial function (**I & J**). (**K**) SOM temporal clustering profiling analysis in aged animals identified 56 DMRs on 16 DEGs with genes also related mitochondria as well as RNA splicing (**L**).
