# Supplementary material for: Repeated Disuse Atrophy Imprints a Molecular Memory in Skeletal Muscle: Transcriptional Resilience in Young Adults and Susceptibility in Aged Muscle
Source: Adv Sci (Weinh). 2026 Feb 25;13(23):e22726. doi: 10.1002/advs.202522726 (PMC13104094; doi:10.1002/advs.202522726)
Supplement: Supplementary file 6 — Supporting File 6: advs74388‐sup‐0006‐Figure S6.pdf. [file ADVS-13-e22726-s001.pdf]

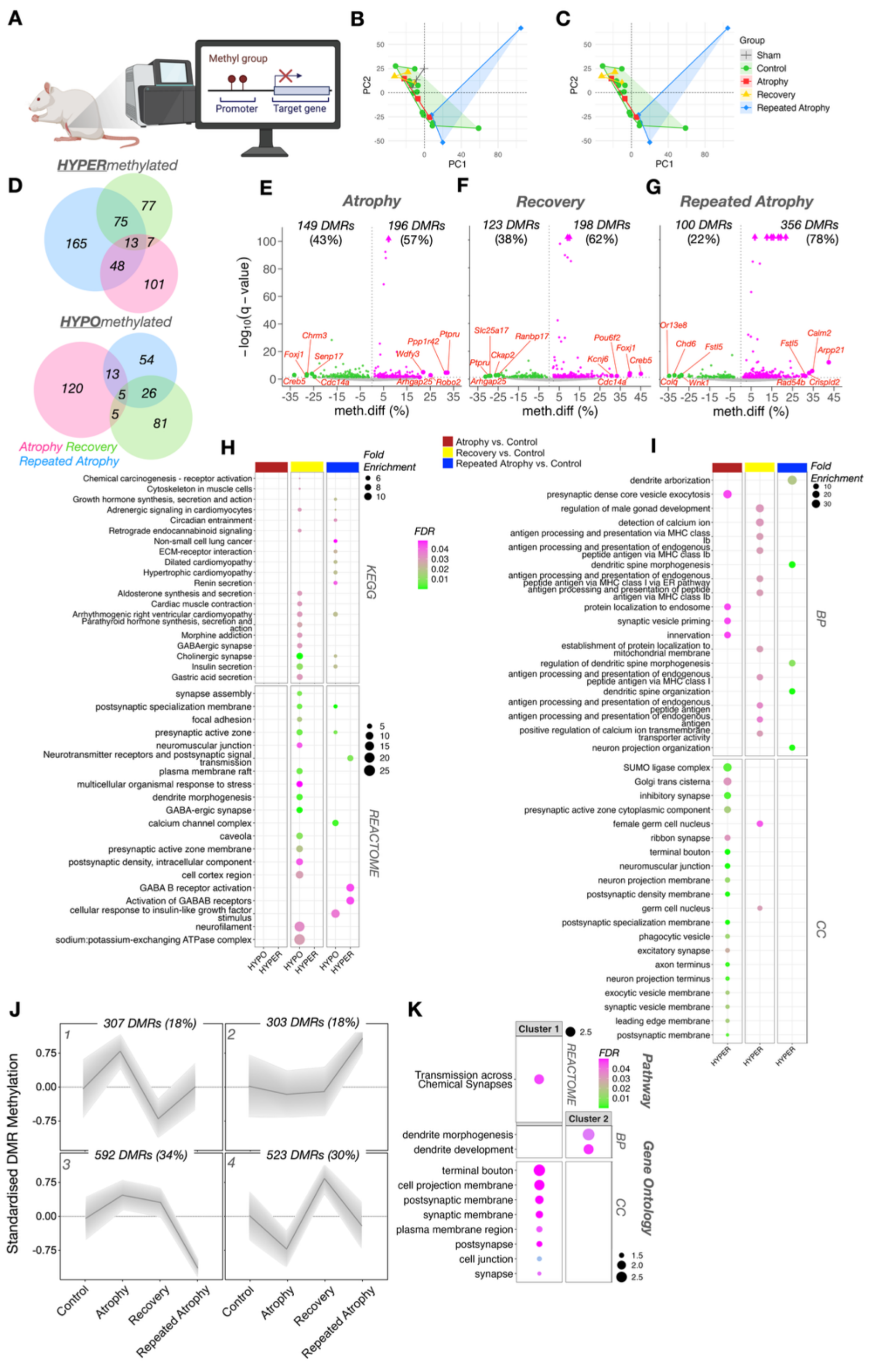

Figure S6. (**A**) Schematic depicting the genome-wide DNA methylation (via Reduced Representation Enzymatic Seq/RREM) response to repeated disuse atrophy in aged rat skeletal muscle (created with BioRender.com). (**B & C**) Principal component analysis (PCA) with clustering by time point, including (**B**) and excluding (**C**) sham control samples. (**D**) Venn diagrams depicting the number of unique and common HYPOmethylated and HYPERmethylated differential methylated regions (DMRs) by gene symbol. (**E-G**) Volcano plots depicting the number of DMRs that were either HYPO-methylated (green) or HYPER-methylated (magenta). Gene symbols within volcano plots represent the top 5 DMRs associated with those genes according to greatest methylation difference (meth.diff %) with a q-value (FDR) ≤ 0.05. Over-representation analysis (ORA) of the top 20 enriched (**H**) pathway (KEGG, top 3 panels; REACTOME, bottom 3 panels) and (**I**) gene ontology (GO) terms for both HYPO-methylated and HYPER-methylated DMRs across the main pairwise comparisons ("Atrophy vs. Control", "Recovery vs. Control", "Repeated Atrophy vs. Control"). (**J**) Self-Organizing Maps (SOM) temporal clustering analysis of 1725 DMRs by gene symbol identified gene clusters with similar temporal profiles across the time course of repeated disuse. (**K**) Pathway and gene ontology (GO) analysis of these SOM cluster profiles revealed most enriched terms related to the neuromuscular junction development and structure and action potential. BP = biological process, CC = cellular component, MF = molecular function.
