# Supplementary material for: Repeated Disuse Atrophy Imprints a Molecular Memory in Skeletal Muscle: Transcriptional Resilience in Young Adults and Susceptibility in Aged Muscle
Source: Adv Sci (Weinh). 2026 Feb 25;13(23):e22726. doi: 10.1002/advs.202522726 (PMC13104094; doi:10.1002/advs.202522726)
Supplement: Supplementary file 7 — Supporting File 7: advs74388‐sup‐0007‐Table S1.pdf. [file ADVS-13-e22726-s002.pdf]

Table S1. Pre-surgical and post-intervention animal body weights (g). Values presented as mean ± standard deviation (SD).

Condition	Pre	Post
Sham/Control<br>*(no TTX)*	374 ± 37	325 ± 102
Atrophy<br>*(6-7 d TTX)*	378 ± 60	357 ± 36
Recovery<br>*(6-7 d TTX + 9 d recovery)*	434 ± 31	392 ± 28
Repeated Atrophy<br>*(6-7 d TTX + 9 d recovery + 5-6 d TTX)*	420 ± 9	344 ± 7*
Mean	402	354**
SD	43	54

TTX, tetrodotoxin. * depicts significant reductions in post- versus pre-body weight in repeated atrophy and mean of all conditions. *$p \leq 0.05$, **$p \leq 0.01$.
