# Supplementary material for: Repeated Disuse Atrophy Imprints a Molecular Memory in Skeletal Muscle: Transcriptional Resilience in Young Adults and Susceptibility in Aged Muscle
Source: Adv Sci (Weinh). 2026 Feb 25;13(23):e22726. doi: 10.1002/advs.202522726 (PMC13104094; doi:10.1002/advs.202522726)
Supplement: Supplementary file 8 — Supporting File 8: advs74388‐sup‐0008‐Table S2.pdf. [file ADVS-13-e22726-s010.pdf]

Table S2. Software and Code Availability

Package name	Version	Link	Reference
openxlsx	v4.2.8	https://CRAN.R-project.org/package=openxlsx	Schauberger P, Walker A (2025). _openxlsx: Read, Write and Edit xlsx Files_.
readxl	v1.4.5	https://CRAN.R-project.org/package=readxl	Wickham H, Bryan J (2025). _readxl: Read Excel Files_.
stringr	v1.5.2	https://CRAN.R-project.org/package=stringr	Wickham H (2025). _stringr: Simple, Consistent Wrappers for Common String Operations_.
dplyr	v1.1.4	https://CRAN.R-project.org/package=dplyr	Wickham H, François R, Henry L, Müller K, Vaughan D (2023). _dplyr: A Grammar of Data Manipulation_.
annotatr (used for RNA-seq)	v1.32.0	https://academic.oup.com/bioinformatics/article/33/15/2381/3092365	Cavalcante RG, Sartor MA. annotatr: genomic regions in context. Bioinformatics. (2017) 33(15):2381-2383. doi:10.1093/bioinformatics/btx183
STAR	v2.7.3a	https://doi.org/10.1093/bioinformatics/bts635	Dobin A*, et al.* STAR: ultrafast universal RNA-seq aligner. *Bioinformatics* **29**, 15-21 (2013)
DeepVenn	Open source-Most recent	https://www.deepvenn.com/	Hulsen T. DeepVenn - a web application for the creation of area-proportional Venn diagrams using the deep learning framework Tensorflow.js. *ArXiv* **abs/2210.04597**, (2022).
DAVID	Open source-Most recent	https://davidbioinformatics.nih.gov/	Huang DW, Sherman BT, Lempicki RA. Systematic and integrative analysis of large gene lists using DAVID bioinformatics resources. *Nature Protocols* **4**, 44-57 (2009).
cowplot	V1.2.0	https://CRAN.R-project.org/package=cowplot	Wilke C (2025). _cowplot: Streamlined Plot Theme and Plot Annotations for 'ggplot2'_.
org.Hs.eg.db	v3.20.0	https://doi.org/10.18129/B9.bioc.org.Hs.eg.db	Carlson M (2024). _org.Hs.eg.db: Genome wide annotation for Human_. R package version 3.20.0.
org.Rn.eg.db	v3.21.0	https://doi.org/doi:10.18129/B9.bioc.org.Rn.eg.db	Carlson M (2024). _org.Rn.eg.db: Genome wide annotation for Rat_. R package version 3.20.0.
ggplot2	v4.0.0	https://ggplot2.tidyverse.org	H. Wickham. ggplot2: Elegant Graphics for Data Analysis. Springer-Verlag New York, 2016.
ComplexHeatmap	v2.22.0	http://bioconductor.org/packages/ComplexHeatmap/	1. Gu, Z. Complex Heatmap Visualization. iMeta 2022.<br>2. Gu, Z. Complex heatmaps reveal patterns and correlations in multidimensional genomic data. Bioinformatics 2016.
circlize	v0.4.16	https://cran.r-project.org/package=circlize	Gu, Z. circlize implements and enhances circular visualization in R. Bioinformatics 2014.
ComplexUpset	v1.3.6	http://doi.org/10.5281/zenodo.3700590	Alexander Lex, Nils Gehlenborg, Hendrik Strobelt, Romain Vuillemot, Hanspeter Pfister, UpSet: Visualization of Intersecting Sets, IEEE Transactions on Visualization and Computer Graphics (InfoVis '14), vol. 20, no.12, pp. 1983–1992, 2014.
enrichplot	v1.26.6	https://doi.org/10.18129/B9.bioc.enrichplot	Yu G (2025). _enrichplot: Visualization of Functional Enrichment Result_. doi:10.18129/B9.bioc.enrichplot
clusterProfiler	v4.14.6	https://yulab-smu.top/contribution-knowledge-mining/	T Wu, E Hu, S Xu, M Chen, P Guo, Z Dai, T Feng, L Zhou, W Tang, L Zhan, X Fu, S Liu, X Bo, and G Yu. clusterProfiler 4.0: A universal enrichment tool for interpreting omics data. The Innovation. 2021, 2(3):100141

DOSE	v4.0.1	https://yulab-smu.top/contribution-knowledge-mining/	Guangchuang Yu, Li-Gen Wang, Guang-Rong Yan, Qing-Yu He. DOSE: an R/Bioconductor package for Disease Ontology Semantic and Enrichment analysis. Bioinformatics. 2015, 31(4):608-609
ReactomePA	v1.50.0	https://yulab-smu.top/contribution-knowledge-mining/	Guangchuang Yu, Qing-Yu He. ReactomePA: an R/Bioconductor package for reactome pathway analysis and visualization. Molecular BioSystems. 2016, 12(2):477-479
enrichplot	v1.26.6	https://yulab-smu.top/contribution-knowledge-mining/	Guangchuang Yu, Li-Gen Wang, Guang-Rong Yan, Qing-Yu He. DOSE: an R/Bioconductor package for Disease Ontology Semantic and Enrichment analysis. Bioinformatics. 2015, 31(4):608-609
TxDb.Rnorvegicus.UCSC.rn6.refGene	v3.4.6	https://doi.org/doi:10.18129/B9.bioc.TxDb.Rnorvegicus.UCSC.rn6.refGene	Team BC, Maintainer BP (2019). _TxDb.Rnorvegicus.UCSC.rn6.refGene: Annotation package for TxDb object(s)_.
TxDb.Hsapiens.UCSC.hg38.knownGene	v3.20.0	10.18129/B9.bioc.TxDb.Hsapiens.UCSC.hg38.knownGene	Team BC, Maintainer BP (2024). _TxDb.Hsapiens.UCSC.hg38.knownGene: Annotation package for TxDb object(s)_.
annotatr (used for methylation)	v1.34.0	https://doi.org/10.18129/B9.bioc.annotatr	Cavalcante RG, Sartor MA (2017). "annotatr: genomic regions in context." Bioinformatics. R package version 1.34.0.
bcl2fastq /2.19.1	v2.19.1	https://support.illumina.com/sequencing/sequencing_software/bcl2fastq-conversion-software.html	https://support.illumina.com/content/dam/illumina-support/documents/downloads/software/bcl2fastq/bcl2fastq-2-19-1-release-notes-1000000035330-00.pdf
umi_tools	v1.1.4 (human) v1.1.6 (rat)	https://doi.org/10.1101/gr.209601.116	Smith T, Heger A, Sudbery I. UMI-tools: modeling sequencing errors in Unique Molecular Identifiers to improve quantification accuracy. Genome Res 27, 491-499 (2017).
trim_galore	v0.6.10	https://doi.org/10.5281/zenodo.5127898	Felix Krueger, Frankie James, Phil Ewels, Ebrahim Afyounian, Michael Weinstein, Benjamin Schuster-Boeckler, Gert Hulselmans, & sclamons. (2023). FelixKrueger/TrimGalore: v0.6.10 - add default decompression path (0.6.10). Zenodo. https://doi.org/10.5281/zenodo.7598955
bismark/0.22.1 - requires- bowtie2; includes- deduplicate_bismark; bismark_methylation_extractor	v0.22.1	https://doi.org/10.1093/bioinformatics/btr167	Krueger F, Andrews SR. Bismark: a flexible aligner and methylation caller for Bisulfite-Seq applications. Bioinformatics 27, 1571-1572 (2011).
bowtie2	v2.4.1	https://github.com/BenLangmead/bowtie2	Langmead B, Salzberg S. Fast gapped-read alignment with Bowtie 2. *Nature Methods*. 2012, 9:357-359.
methylKit -includes calculateDiffMeth; percMethylation	v1.32.1	http://doi.org/10.18129/B9.bioc.methylKit	Altuna Akalin, Matthias Kormaksson, Sheng Li, Francine E Garrett-Bakelman, Maria E Figueroa, Ari Melnick and Christopher E Mason. methylKit: a comprehensive R package for the analysis of genome-wide DNA methylation profiles. Genome Biology 13:R87 (2012).
